# The role of gold atom concentration in the formation of Cu–Au nanoparticles from the gas phase

**DOI:** 10.3762/bjnano.12.6

**Published:** 2021-01-19

**Authors:** Yuri Ya Gafner, Svetlana L Gafner, Darya A Ryzkova, Andrey V Nomoev

**Affiliations:** 1 Katanov Khakas State University, Physic Department, Lenin pr. 90, Abakan, 655017, Russia; 2 Institute of Physical Materials Science, Siberian Branch of the Russian Academy of Sciences, Sakhyanova str., 6, Ulan-Ude 670047, Russia

**Keywords:** binary nanoparticles, computer simulation, copper, gold, molecular dynamics

## Abstract

The synthesis of bimetallic nanoparticles need to be controlled in order to obtain particles of a desired size, spatial structure, and chemical composition. In the synthesis of the Cu–Au nanoparticles studied here, nanoparticles can be obtained through either chemical or physical methods, each of which has its own drawbacks. Although it is very difficult to achieve the required target chemical composition of nanoparticles during chemical synthesis, their size can be stabilized quite well. In turn, physical synthesis methods mainly allow to maintain the required chemical composition; however, the size of the resulting particles varies significantly. To solve this issue, we studied the formation of Cu–Au nanoparticles with different chemical compositions from a gaseous medium using computer molecular dynamics (MD) simulation. The aim was to determine the effect of the concentration of gold atoms on the size and on the actual chemical composition of the formed bimetallic nanoparticles. The modeled region had a cubic shape with a face length of 1350 Bohr radii and contained a total of 91125 copper and gold atoms uniformly distributed in space. Thus, based on the results of the MD simulation, it was concluded that an increase in the percentage of gold atoms in the initial vapor phase led to a decrease in the size of the synthesized nanoparticles. In addition, it was found that clusters with a size of more than 400–500 atoms, regardless of the chemical composition of the initial vapor phase, basically corresponded to a given target composition.

## Introduction

The nanometer-sized Cu–Au compounds are being studied quite actively [1–8] because of their potential application in the field of catalysis and optics. Thus, it has been shown that, in heterogeneous catalysis, Cu–Au nanoalloys usually exhibit a synergistic effect in oxidative reactions and the preferential oxidation of carbon monoxide (CO) to carbon dioxide at ambient temperature as well as in the selective oxidation of benzyl alcohol to benzaldehyde and in the epoxidation of propene with nitrous oxide. Moreover, in experimental and theoretical works [9], it was clearly shown that the partial substitution of copper with gold leads to a change in the physicochemical properties in many cases. For example, there is an increase in the catalytic activity when compared to the monometallic Au or Cu nanoparticles [3]. In addition to catalysis, Cu–Au nanoalloys are also of great interest in optics, in which the doping of copper into gold nanoparticles causes the appearance of strong surface plasmon resonances. In this case, the energy and resonance line width depend on the percentage of copper.

A Cu–Au nanoalloy is often produced using standard chemical methods. However, nanoparticles synthesized by these methods usually vary greatly in size, in the percentage of chemical elements, or in the structure formed during chemical reactions. Therefore, the relationship between the initial experimental conditions and the final result is far from explicit [2]. This fact limits the design of Cu–Au nanoparticles for various potential applications and, at the same time, contributes to their study as a model system. In recent years, a wide range of chemical methods have been successfully implemented for the synthesis of Cu–Au nanosystems of various percentages, the use of which led to the production of Cu–Au nanoparticles with spherical [4], pentagonal [5], rod-like [6–7], and cubic [8] shapes. Nanocrystals of a gold and copper alloy ranging from 45 to 200 nm in size and with pronounced pentagonal structures were synthesized in [5] by the joint reduction of gold and copper salts. Here, elemental mapping of Cu and Au, using scanning transmission electron microscopy–energy-dispersive X-ray spectroscopy, confirms the formation of ordered nanosized alloys with a total gold content of 87.4%. In a previous work [8], cubic Cu–Au nanoparticles were obtained through the polyol reduction method with a good control of the size of the resulting clusters. The authors of [8] argued that in the case of Cu_3_Au and Au_3_Cu compositions, they were able to stabilize the edge length of the synthesized nanoparticles with a value ranging from 3.4 to 85 nm.

Although a fairly good control of shape and size can be achieved through chemical methods, it is still unclear whether the chemical composition of the particles can also be finely tuned. This fact is explained by the lack of systematic studies to determine the composition of chemically grown Cu–Au nanoparticles, at the level of single particles, to distinguish between real and nominal compositions obtained in a large ensemble of synthesized clusters. It was experimentally found that, during chemical synthesis, the preferred internal ordering and composition of the nanoparticles of the Cu–Au alloy, even at room temperature, is a very complex process that depends on the particle size and on the environmental properties [10].

Compared to chemical methods, physical methods offer a better control over the composition of the synthesized bimetallic nanoparticles. The fabrication of Cu–Au bimetallic nanoparticles using ion implantation [11] or thermal evaporation in ultrahigh vacuum [12–13] are typical examples of these techniques. Also, in [14], the Cu–Au nanoalloys were deposited onto amorphous carbon or magnesium oxide substrates by the laser evaporation of a bulk alloy with various stoichiometric compositions (Cu–Au, Cu_3_Au, and Au_3_Cu). An analysis of individual clusters carried out by using electron diffraction and high-resolution transmission electron microscopy (HRTEM) showed that Cu–Au clusters are formed with chemical compositions corresponding to the composition of the evaporated material [14]. In the case of cluster deposition onto amorphous carbon, various cluster morphologies were observed, such as cuboctahedral and decahedral. For clusters supported on a MgO substrate, only particles with a truncated octahedral structure were observed. As it can be seen from this work, a high control of the composition can be achieved with the help of physical methods; however, with a caveat that there is no dimensional or morphological homogeneity.

Thus, in all the experiments performed, different external and internal structures of Cu–Au nanoparticles were observed without a clear understanding of which structure, shape, and chemical composition could be formed as a result of one or another synthesis method. Therefore, in the present work we will concentrate on the analysis using computer simulation methods of only one of the physical methods used for the production of binary Cu–Au nanoparticles (i.e, the synthesis from a high-temperature gas phase). For the first time, a detailed study of the dependence of the atomic ordering of Cu–Au particles on the concentration of gold atoms in a sufficiently voluminous model medium is carried out based on a many-particle tight-binding potential, which is quite complex for modeling large systems. Based on the obtained data, we draw conclusions about the features of the formation of such particles, their real chemical composition and internal structure, and the main mechanisms involved in the formation of binary Cu–Au nanoparticles upon deposition from a gaseous medium.

## Computer Model

Synthesis from the gas phase is one of the main physical methods used for producing nanopowders. However, since particle formation reactions occur, as a rule, within nanoseconds and the sizes of the joining initial fragments are in the nanometer range, an experimental analysis of the processes occurring in this case is rather difficult. The theoretical description of the condensation process is also a difficult task, since particles are formed under clear nonequilibrium conditions with a dependence on the physicochemical properties of the nanoparticle determined by its size. In this paper, we present the results of a computer simulation study of the condensation process of gold and copper atoms. For the modeling, we used the molecular dynamics (MD) method, which allows us to analyze the behavior of the simulated system over a wide time interval (from picoseconds to several nanoseconds) and with the accuracy required to consider small atomic groups.

This type of modeling can only be successful if a sufficiently realistic potential of interatomic interaction is used. Since the simulation of the evolution of relatively large systems (several thousands of atoms) in the nanosecond range by quantum mechanical methods is still very difficult, we used a simpler approach based on the modified TB-SMA tight-binding potential [15] with an interaction radius of up to (and including) the fifth coordination sphere. Based on this model, we focused on determining the influence of several initial conditions on the initial stage of the synthesis by condensation of binary Cu–Au nanoparticles. In particular, we focused on the growth processes of these nanoparticles and on the analysis of the chemical composition of the resulting nanoclusters.

The modeled region had a cubic shape with a face length of 1350 Bohr radii and contained a total of 91125 copper and gold atoms uniformly distributed in space. The initial temperature was set to *T*_i_ = 1000 K, which corresponds to the stage of transporting the atoms from the evaporated substances using the buffer gas to the synthesis chamber. The equations of atomic motion were solved based on the Verlet velocity scheme [16] with an integration step of *h* = 1 fs. During nucleation and further growth of the particles from the gaseous medium, the material undergoes dramatic changes in its chemical environment. It is quite problematic to use the inert gas pressure and its effect on the cooling rate of the main gas mixture (i.e., the parameters that are used in a real experiment) in a computer simulation. As a rule, even in a high-density buffer gas, collisions between atoms of the synthesized material occur in the nanosecond range, which is the maximum possible time interval for a MD simulation. Based on this, the analysis we present is only an attempt, at a qualitative level, to understand the main features of the formation of Cu–Au nanoparticles under conditions that are similar to the process of a real synthesis by the condensation of the gas phase.

In our model, all the atoms were randomly distributed in a simple cubic lattice, with an average distance of 30 Bohr radii between them. This was done in order to completely exclude their interaction at the very beginning of the simulation and to achieve a random placement in the synthesis zone. The inert gas atoms cooling the main simulated gas mixture were set using the Andersen thermostat [16] with a control parameter *W*, in which the total system energy is calculated as:


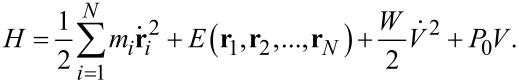


According to this stochastic method, the metal atoms undergo random collisions with certain virtual particles to model the interaction with the atoms of the cooling inert gas. The temperature of the cooling inert gas, under the adopted experimental conditions, corresponded to the temperature of liquid nitrogen.

In our model, the inert gas flow rate was set by the rate of thermal energy removal. However, since this effect is well enough understood, it was not considered in the present work and the simulation was conducted with only one cooling rate. Therefore, the main objective of the study was to determine the possible effect of the initial chemical composition of the gas phase, in the synthesis chamber, on the size distribution of the final particles and on the chemical composition of gold and copper atoms. To analyze the processes occurring during condensation, four different target compositions were considered: CuAu, Cu_60_Au_40_, Cu_3_Au, and Cu_90_Au_10_ with a fairly uniform decrease in the percentage of gold atoms in them. A more complete description of the approach used to simulate the condensation process was previously given in [17]. The computer program called MDNTP, developed by Dr. Ralf Meyer at the University of Duisburg (Germany), was used for the MD simulations.

## Results and Discussion

During the simulation of the condensation process of copper and gold atoms, the temperature in the synthesis region was determined by two independent factors: the cooling process of the Cu–Au atomic vapor by a buffer gas and the release of the thermal energy converted from the binding energy of the atoms [18–19]. The cooling rate of the atomic vapor was not directly controlled during the simulation. In fact, it was determined by the collision frequency and by the difference between the mass values of the atoms of the cooling substance and the metal atoms. In our model, the buffer gas was the gas consisting of virtual particles created by the Andersen thermostat. This technique allows one to quickly remove the excess energy from the system through elastic collisions between virtual particles and metal atoms. The chosen cooling rate of gold and copper atoms was *U* = 1 × 10^10^ K/s, and the cooling process was carried out at *T*_f_ = 77 K.

Initially, to determine the degree of reliability of the obtained results, a direct comparison was made between the data obtained from our MD simulation and the data on the size distribution of Cu_3_Au clusters obtained by laser deposition [3]. An analysis of the shape and distribution of the Cu_3_Au clusters on the substrate indicates that the agglomeration processes were suppressed in this case [3]. The reason for that may be the wide spatial distribution of the evaporated primary fragments of the cluster due to the high ambient temperature. This interferes with the combination of the resulting clusters as a result of the high kinetic energy of the atoms, and, possibly, also of the short time of approach to the substrate. As a result, in the case of Cu_3_Au, spherical nanoparticles well separated in space were obtained with an average size of 1.9 ± 0.7 nm [3]. The maximum possible cluster size was 5.5 nm. The nanoparticles were single crystals with the face-centered cubic (FCC) structure displayed along the [110] axis and their lattice parameters were determined from 15 particles obtained from HRTEM images. From these measurements, the average lattice parameter of the synthesized Cu_3_Au nanoparticles was estimated to be 3.74 ± 0.01 Å. The fact that this value lies between the lattice parameters values of pure gold (*a*_Au_ = 4.078 Å) and pure copper (*a*_Cu_ = 3.610 Å) is an evidence that the nanoparticles deposited onto the amorphous carbon substrate were a Cu–Au nanoalloy [3].

Next, we analyzed the structure of the Cu_3_Au nanoparticles [3] in order to determine the possible temperature of the nanoparticles at the moment of their collision with the substrate. As shown in our previous work [20], when the crystallization takes places outside the substrate, spherical particles of a Cu–Au nanoalloy of this size in most cases have a pronounced five-fold structure (icosahedral or decahedral); nonetheless, the FCC structure was quite rare. However, when laser deposition was used, the nanoparticles found in [3] exhibited the FCC structure. In this case, during the deposition, the substrate was bombarded with liquid droplets of a Cu_3_Au nanoalloy. Once at the substrate, they spread out and acquired a round shape. As a result, after crystallization the FCC structure was formed. The formation of the FCC structure with such a small cluster size in all synthesized Cu_3_Au nanoparticles can only be explained by the fact that, at the moment of crystallization, the clusters were not in a state of free motion and, therefore, could not have a spherical 3D shape corresponding to a minimum of surface energy. Therefore, we can conclude that the Cu_3_Au clusters precisely hit the substrate in the liquid state through collision, which corroborates the HRTEM image of a flat 2D nanoparticle [3]. Since particles with a maximum size of 5.5 nm (approx. 7000 atoms) were found in the spatial distribution shown in [3] and a pure copper cluster melts at 1100 K [21], even with a size of 2200 atoms, the temperature of the system should be equivalent to the higher melting point (*T*_m_ = 1358 K for copper). Therefore, a comparative analysis between the spatial distribution shown in [3] and our MD simulation was carried out at a simulation time of *t* = 5.5 ns. At that simulation time, the temperature of the simulated space was still high (*T* = 1380 K) and an active cluster formation in the liquid state has already been observed.

**Figure 1:**
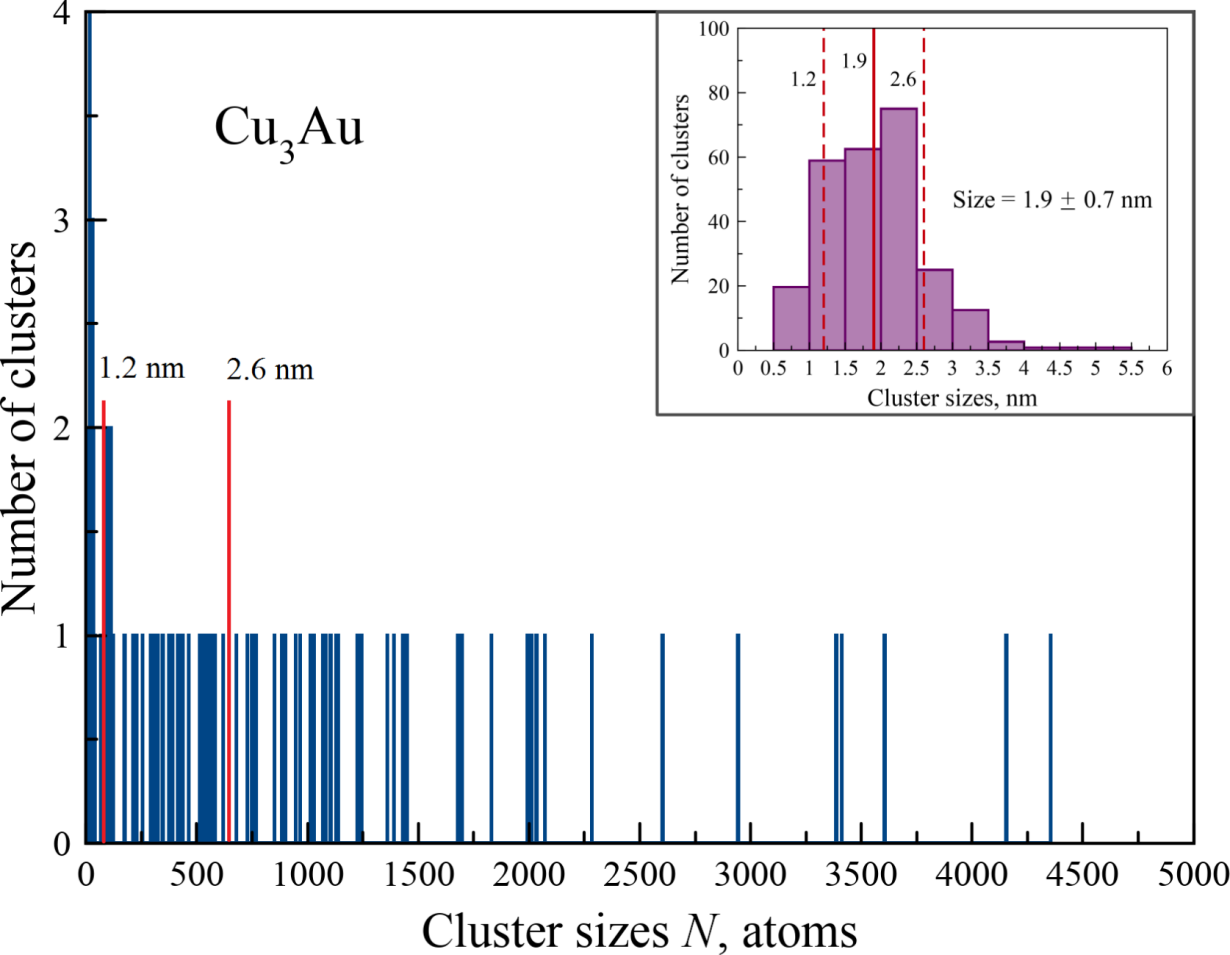
Size distribution of binary nanoparticles with the target composition of Cu_3_Au at a temperature of *T* = 1380 K. The MD simulation time is *t* = 5.5 ns. The inset shows the experimental size distribution based on the data presented in [3] for Cu_3_Au nanoparticles obtained by laser deposition.

According to Figure 1, at this temperature most clusters have a size of approx. 250–600 atoms, which corresponds to the diameter of the spherical cluster (*D* = 1.8–2.5 nm). This practically coincides with the data shown in [3] (*D* = 1.9 ± 0.7 nm, indicated in the inset of Figure 1). The only difference is that we still observed quite a few single atoms (*N* = 44), dimers (*N* = 219), trimers (*N* = 71), and cluster fragments of a very small size (i.e., consisting of 4–10 atoms, *N* = 134), which were simply impossible to see on the HRTEM images [3]. Therefore, for convenience, small cluster fragments with sizes of up to 15 atoms were not shown in Figure 1. The value of the maximum cluster recorded during the MD modeling was slightly lower (4370 atoms) than that of [3]. This cluster size corresponds to the diameter of approx. 4.76 nm in the case of a spherical cluster. In fact, the nanoparticle with the largest number of atoms (Figure 1) had a linear shape, obtained by the agglomeration of many primary clusters. Therefore, its true size can be much larger. Consequently, we can conclude that with the chosen simulation parameters (i.e., the size of the system and the number of atoms contained in it) it was possible to repeat the results obtained from direct synthesis experiments of Cu–Au nanoparticles with a fairly good degree of reliability.

Next, we performed a detailed analysis of the results obtained during the MD modeling. For a more accurate understanding of all the processes, we first provide data on copper condensation since gold atoms were considered as an impurity in this case. In part, we performed the analysis shown in [22], which shows that under similar conditions of simulation synthesis, at the final stage of the simulation (*T* = 77 K), most of the particles obtained had a size of up to 4000 atoms. This value corresponds to the approximate diameter value of the spherical cluster (*D* = 4.61 nm). In addition to this basic group, another five particles with a size of up to 17000 atoms were discovered, which were formed by agglomeration at sufficiently low temperatures. We observed a similar pattern for all of the heat energy removal rates used. Therefore, this distribution can be considered basic taking into account that with an increase in the cooling rate, the maximum cluster size is expected to decrease in the simulated system, in full accordance with the experimental data [18].

**Figure 2:**
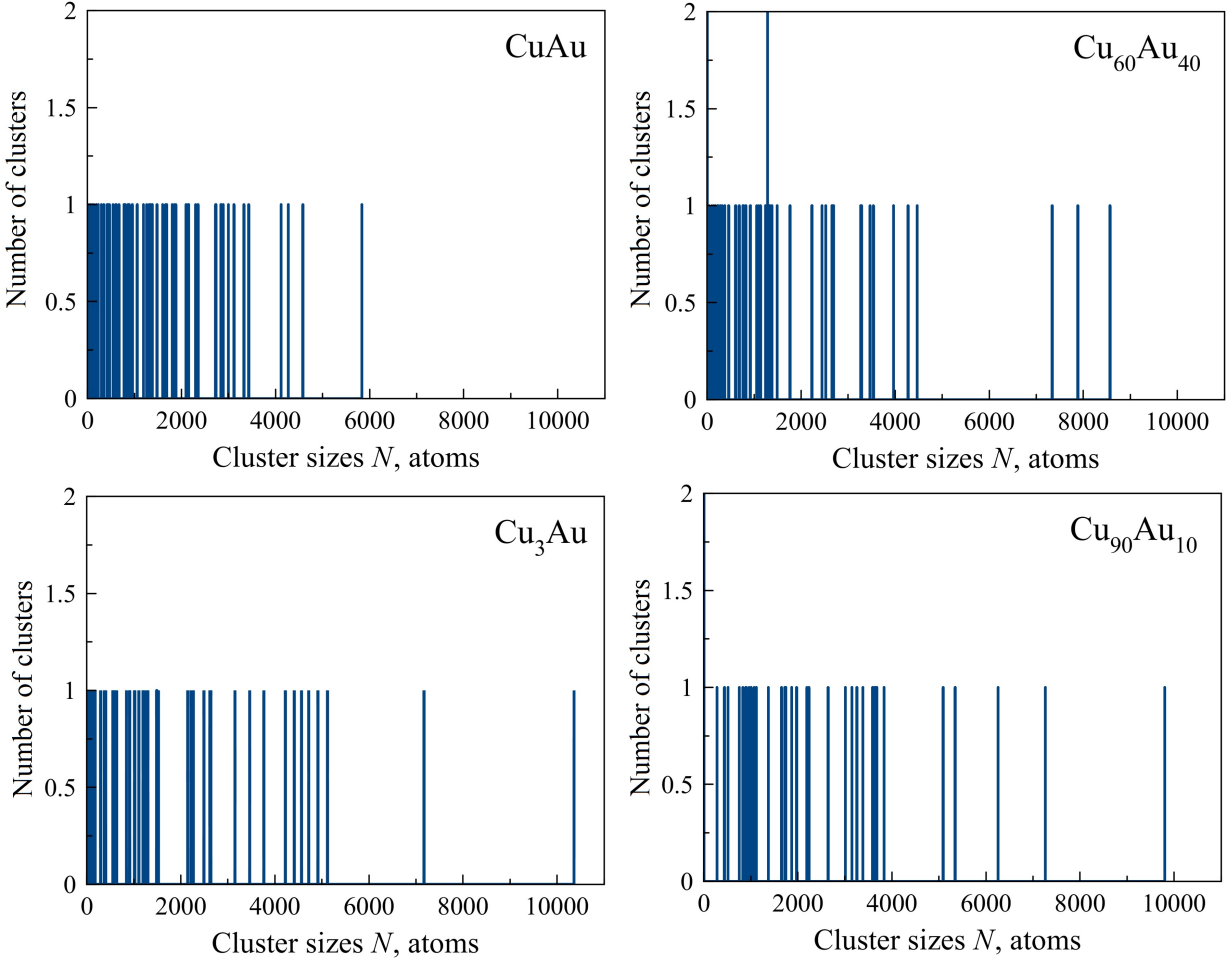
Size distribution of binary Cu–Au nanoparticles with different chemical compositions at *T* = 77 K.

In order to verify this, we compared these results with the data from the performed MD simulation for the Cu_90_Au_10_ case. A size distribution very similar to that of the simulation results of Cu nanoparticles is seen for binary Cu–Au particles obtained from this chemical composition of the initial gas medium. The main group containing clusters with a size up to 4000 atoms and five larger particles were also recorded; nonetheless, the largest cluster was smaller (Figure 2). However, with an increase in the percentage of gold atoms in the initial binary vapor, the situation significantly changes. It is noticeable that, in this case, there is a decrease in the maximum cluster size as well as in the number of clusters with a relatively large size (Figure 2). This means that there is a clear tendency to suppress the agglomeration of nanoparticles by increasing their content of gold atoms.

It is known that in the bulk state, the melting points of copper and gold are practically equal (*T*_m_ = 1358 K and *T*_m_ = 1337 K, respectively). However, this is no longer true in the nanoscale case. The MD modeling [21,23] showed that at an equal size (*D* < 10 nm), the melting temperature of copper nanoclusters is 30–40% higher than that of gold clusters, which means a lower binding energy between gold atoms in these small clusters. This is, most likely, decisive for the formation of binary Cu–Au nanoparticles. In the initial stages of formation, due to the high temperature of the simulated system, Cu–Au clusters are small drops of a highly heated liquid. To reduce the surface energy of the liquid droplets when they are combined, it is often energetically advantageous to separate them again into several parts. It is clear that the lower the binding energy between the atoms, the greater the extent to which these processes will occur, as observed in the case in which a higher percentage of gold atoms in Cu–Au nanoparticles is used.

The effect we found in a computer MD modeling based on the tight-binding potential has a direct experimental confirmation. In [3], a comparative synthesis of Au_3_Cu and Au nanoparticles was performed using a chemical method and a laser evaporation method. In [3], the laser energy and the number of necessary laser shots for the evaporation of each metal sample were presented. After analyzing the data, one can notice that the laser energy required to evaporate gold is approx. 100 mJ lower than the energy required to evaporate copper. Also, despite the lower laser energy used, the rate of evaporation was much higher for gold than for copper. As a result, the required number of laser shots to evaporate the gold sample was much lower than that to evaporate the copper sample.

Besides, it was found in [9] that during the heating of Cu_3_Au nanoclusters, typical changes called “order–disorder transition” occur in the nanocluster structure, leading to the decomposition of the initial L1_2_ crystal structure. This leads to a transition to the FCC structure with a random atomic distribution in the long-range coordination spheres. In the case of bulk, copper and gold atoms are distributed arbitrarily at the sites of the face-centered cubic lattice. However, it was shown in [9] that with an increase in the temperature, the initially homogeneous chemical composition of Cu–Au nanoparticles started to change due to the displacement of Au atoms into the surface layer. Moreover, the rate of segregation increased with temperature, which was experimentally confirmed [24].

This effect is a direct consequence of the different binding energies of gold and copper atoms (i.e., with these nanoparticle sizes, the binding energy of copper atoms is higher than that of gold atoms). Consequently, the more copper atoms in a binary Cu–Au nanoparticle, the greater the probability of preserving the primary clusters joined in a random motion in a single nanoparticle (Figure 2). An analysis of the chemical composition of Au_3_Cu and Cu_3_Au performed in [3] shows that the average nanoparticle size was 4.8 ± 1.3 nm in the Au_3_Cu sample, while the Cu_3_Au nanoparticles had an average size of 22.9 ± 1.5 nm. This experimental result confirms our conclusion that an increase in the content of gold atoms in Cu–Au nanoparticles leads to a suppression of the extent of their agglomeration processes.

Next, we considered the effect of the initial content of gold atoms in a gaseous medium composed of Cu–Au on the chemical composition of the synthesized nanoparticles. It is known that a characteristic feature of chemical synthesis techniques is the heterogeneity in the composition of the resulting nanoparticles. Thus, the experimental results in [3] showed that in nanoparticles with a nominal composition of Au_3_Cu, a random change in the content of copper atoms was found, without any explicit size dependence. The Cu content ranged from 6 to 23 atom % in binary particles with sizes up to 8 nm and with an average composition of 11 ± 3 atom % Cu.

A similar analysis undertaken for nanoparticles with a target composition of Cu_3_Au also showed the absence of a clear relationship between their size and composition. Here, the chemical composition was between 8 and 19 atom % Cu with an average composition of 12 ± 2 atom % Cu for nanoparticles with sizes up to 26 nm. Also, the formation of small spherical particles with an average size of approx. 2 nm, which seemed to surround a much larger nanoparticle with a nominal composition of Cu_3_Au, was noticed in [3]. An experimental study of the ensemble of these nuclei demonstrated that they are nanostructures consisting of pure copper.

The obtained results clearly illustrate that the spectral analysis, often used to determine the chemical composition of sufficiently large samples, can take into account not only the actual composition of Cu–Au nanoparticles but also include the composition of all very small copper particles, which leads to a reassessment of the real copper content in the particles. Therefore, it is very important to measure the composition of the synthesized nanoparticles pointwise to exclude the contribution of the small Cu clusters.

Incomplete doping of gold in Cu–Au nanoparticles can be explained based on the chemical reactions that take place in [3]. As shown in [25], the redox potential of (Au^3+^/Au) is +1.50 V. In the case of Cu, the corresponding potential is only +0.34 V [26]. Therefore, gold ions are more easily reduced in a gold sample than copper ions in a copper bulk. Therefore, gold nuclei can be formed before all the copper ions are reduced. The copper atoms that remain after the chemical reaction form small Cu nanoparticles, as observed in the experiments shown in [3].

In the case of physical methods for producing Cu–Au nanoparticles, it seems possible to create particles with the desired chemical composition. In [3], the Cu–Au nanoparticles obtained by laser deposition on a graphite substrate were analyzed via transmission electron microscopy–energy-dispersive X-ray spectroscopy (TEM-EDS). Based on the analysis of the spectra, the average of Cu content was 20 ± 1 atom % in the sample with the target composition of Au_3_Cu and 73 ± 3 atom % in the sample with the target composition of Cu_3_Au. Also, a MD modeling based on bond-order potential simulated the synthesis of SiC_2_ nanoparticles from a gaseous medium [2]. The formation of SiC_2_ clusters was studied at different initial concentrations of atoms and the particles obtained at the final stage (simulation time of 5 ns) mainly had a stoichiometric composition.

**Figure 3:**
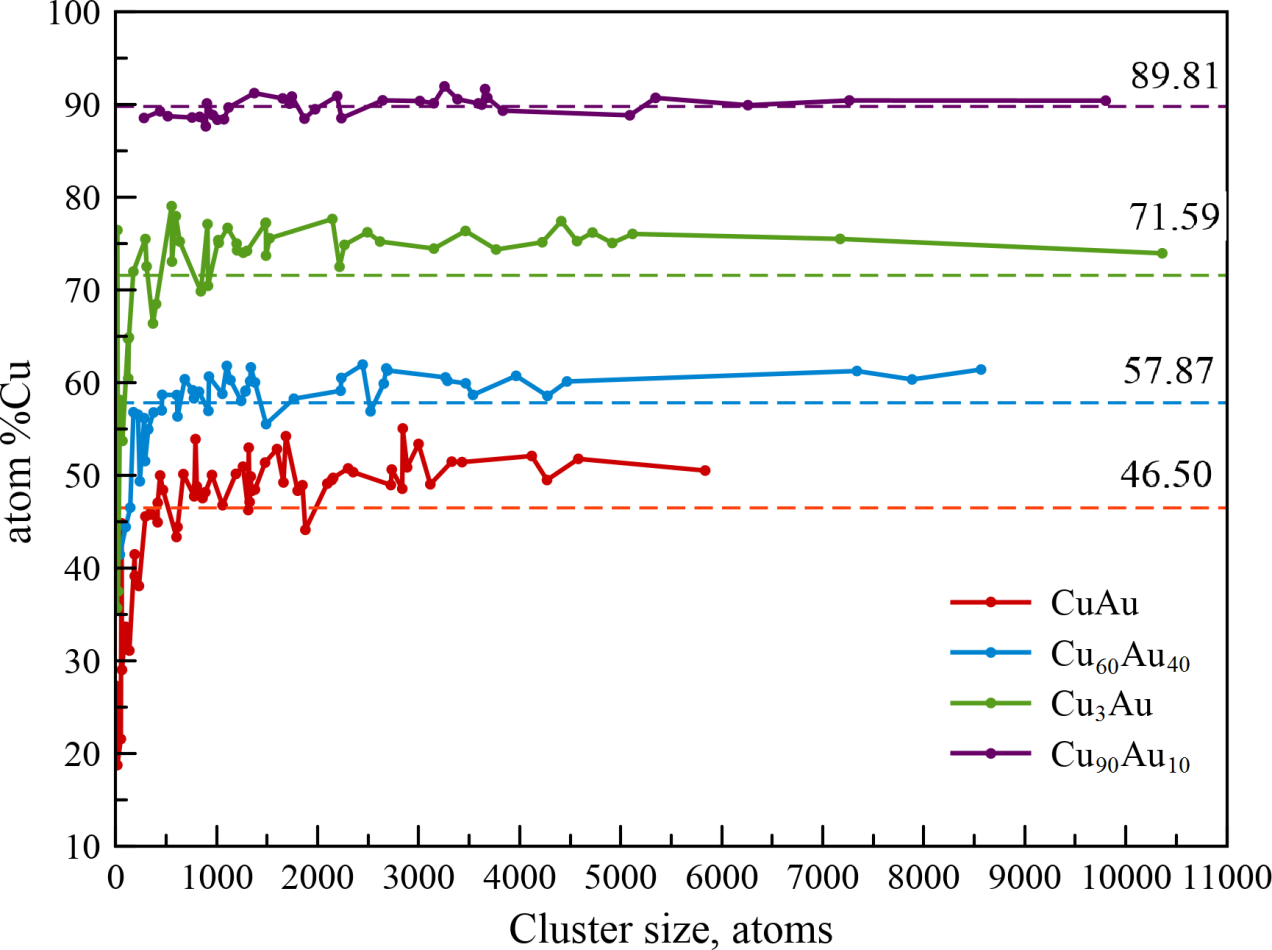
The percentage of copper atoms in binary Cu–Au nanoparticles with different chemical compositions of the initial gas mixture. The analysis was carried out at the final stage of the simulation (*T* = 77 K, *t* = 25 ns). Dashed lines show the average content of copper atoms in binary Cu–Au nanoparticles at various target compositions.

Figure 3 shows the data on the percentage of copper atoms in all the clusters with the following target compositions: Cu_50_Au_50_, Cu_60_Au_40_, Cu_75_Au_25_, and Cu_90_Au_10_ obtained in our MD modeling. The average content of copper atoms was quite close to the required values: 46.50 atom % for the target composition of 50% (deviation of 3.50%), 57.87 atom % for the target composition of 60% (deviation of 2.13%), 71.59 atom % for the target composition of 75% (deviation of 3.41%), and 89.81 atom % for the target composition of 90% (deviation of 0.19%). Besides, our computer simulation results on the synthesis of binary Cu–Au nanoparticles from a gaseous medium are in good agreement with the experimental data for the Cu_3_Au (73 ± 3 atom %) case shown in [3].

**Figure 4:**
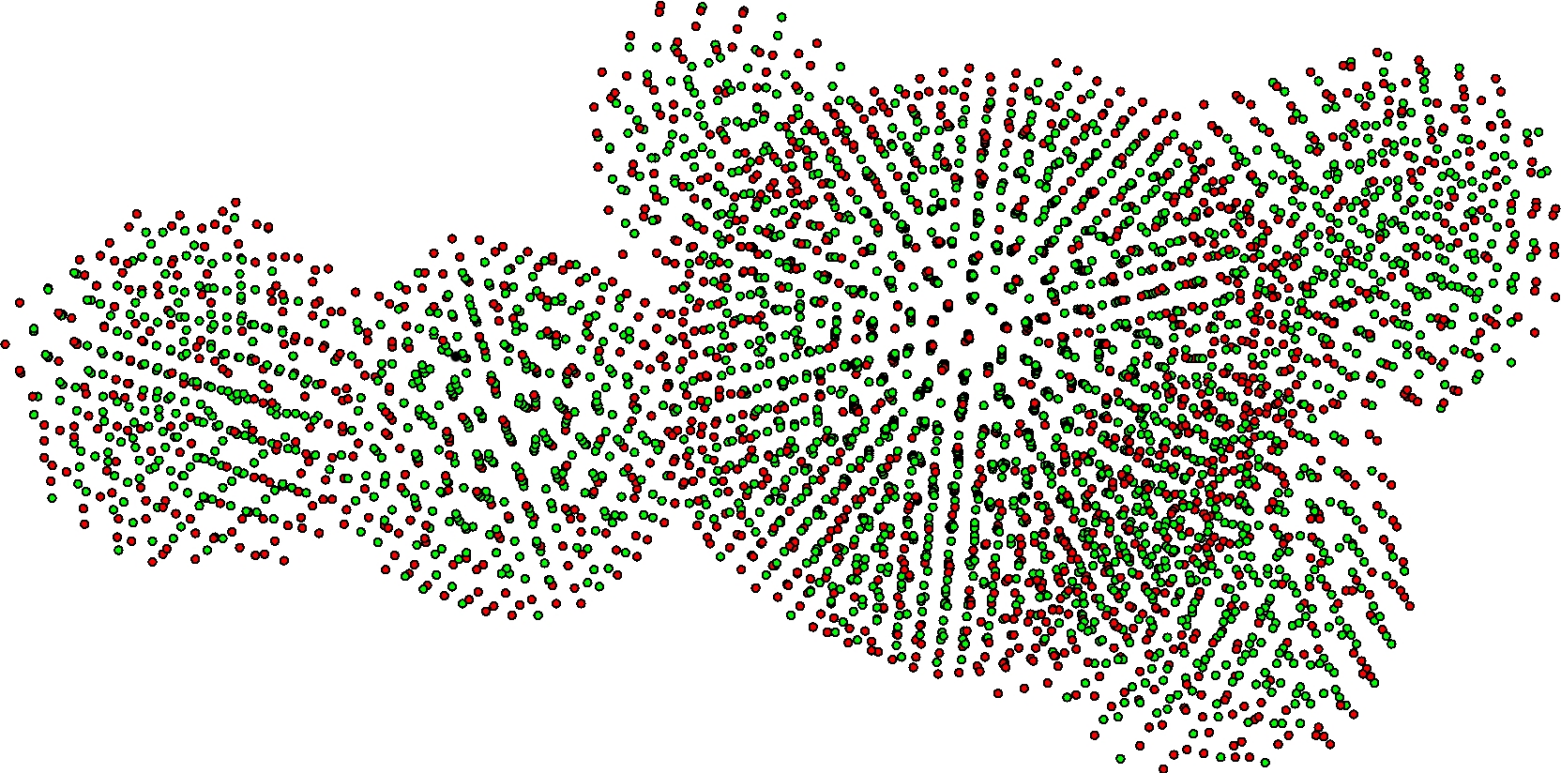
Image of a Cu_60_Au_40_ nanoparticle obtained by MD modeling. Copper atoms are shown in green and gold atoms are shown in red.

Even though Cu–Au nanoclusters with a five-fold symmetry were chemically synthesized [5], it is possible to obtain Cu–Au nanoclusters with the usual FCC structure using these methods. However, as shown in the results of our MD simulation, the arrangement of atoms in many Cu–Au nanoparticles precisely corresponded to a five-fold symmetry (Figure 4). Thus, we can conclude that the physical methods used for the synthesis of binary Cu–Au particles contribute to the formation of icosahedral clusters in contrast to the chemical methods, in which those processes are difficult to happen. The appearance of the particle in Figure 4 indicates that its formation occurred at low temperatures. This assumption is confirmed by the fact that, at these temperatures, the process of coalescence (fusion) of the clusters among themselves is almost completed due to the lack of the high kinetic energy necessary for this. In addition, the agglomeration processes, or simply particles sticking together, begin to prevail as shown in Figure 2. Therefore, the formation of an icosahedral structure had a nature different from coalescence. In [27], we suggested and verified the assumption that a lower density of a nanoscale compact material, in comparison with ordinary bulk samples, leads to an increase in the probability of formation of an atomic structure with a five-particle symmetry due to the greater diffusion mobility of the atoms in a nanoscale compact material. A similar effect can be observed in this case.

According to Vegard’s law, the lattice parameter of bimetallic alloys varies linearly, depending on the composition, between the lattice parameters of the pure components. In the framework of the experiments shown in [3], its validity was also confirmed for the Cu–Au bimetallic nanoalloy case. The sample consisting of 20 ± 1 atom % Cu had an average lattice parameter of 3.99 ± 0.01 Å and, for a sample consisting of 73 ± 3 atom % Cu, the average lattice parameter was 3.74 ± 0.01 Å. For comparison, the lattice parameter of pure copper is 3.610 Å and that of pure gold is 4.078 Å. Thus, the introduction of gold atoms into a copper lattice leads to its loosening, which in the case of nanoclusters, allows copper atoms to more easily diffuse. In a sufficiently long time, and in our case the real-time of the MD simulation was 25 ns, this circumstance can enable the formation of an icosahedral structure [22].

However, in the case of stoichiometric compositions of CuAu and Cu_3_Au, these processes are influenced by crystallographic laws, which require the rearrangement of the atoms into an L1_0_ or L1_2_ superstructure. In addition, the displacement of gold atoms to the cluster surface also plays a role [9]. A cluster begins to form with a copper core and a gold shell (core–shell clusters). The surface atoms already have a lower binding energy due to a decrease in their coordination number, and surface gold atoms have an even lower binding energy when compared to the atoms of the copper center.

At high temperature values in the synthesis chamber, the influence of all these factors prove that the discharge of the smallest droplets of gold from the surface of a binary cluster can be energetically favorable. Note that the copper content in very small clusters obtained in the synthesis with the target composition of Cu_3_Au (approx.18%) is comparable with the results show in [3]. However, this is only valid when comparing with the chemical production method (11–12 atom % Cu). This gives us reason to believe that a different mechanism for the formation of a binary compound was involved here when compared to clusters of a larger size (*N* > 400–500 atoms) which were conditionally formed according to the “physical” scenario. Apparently, at such small sizes, the incomplete content of copper in Cu_3_Au nanoparticles can be explained only based on the kinetics of chemical reactions.

Thus, according to the MD simulation results, several important conclusions can be drawn. First, an increase in the percentage of gold atoms in a binary vapor composed of Cu–Au leads to a decrease in the size of the synthesized nanoparticles. Second, very small clusters with a high content of gold atoms are observed. With an increase in the cluster size, this effect decreased and, then, completely disappeared. Third, clusters with a size of more than 400–500 atoms, regardless of the chemical composition of the initial vapor, mainly adhere to the specified target composition.

## Conclusion

In recent years, the study of nanomaterials is one of the fastest growing areas of scientific knowledge. In scientific and technological fields, there is a special interest in studying metal binary nanoparticles among a wide range of nanosystems. These particles usually exhibit new chemical and physical properties in comparison to conventional bulk materials due to the much higher surface-to-volume ratio. Even these unusual properties of the metal nanoparticles can be changed and fine-tuned by adjusting their size, shape, and chemical composition. Thus, several studies on the composition-dependent control of the metal nanoparticle properties showed that even the combination of two metals into a single nanoparticle (i.e., the formation of a bimetallic nanoparticle or the so-called nanoalloy) can lead to very important synergistic effects in areas such as catalysis of industrial reactions, optics, optoelectronics, and medical applications.

We also noted that predicting the structure of nanoparticles consisting of different chemical elements is not a trivial task because of its dependence on both the size and the complex energy landscape. To achieve equilibrium, bimetallic nanoparticles must optimize both the geometric shape and the chemical ordering. However, nanoparticles grown by a gas-phase synthesis process often fall into metastable configurations due to nonequilibrium growth conditions. Therefore, their configuration is determined not only by energy effects but also by kinetic effects. By using molecular dynamics methods, we tried to determine the chemical ordering of Cu–Au particles in these synthesis processes and to elucidate some of their formation mechanisms. In particular, it was found that an increase in the relative number of gold atoms in the initial gas mixture leads to a decrease in the size of the synthesized nanoparticles. It was also noted that the nanoparticles obtained by condensation from the gas phase corresponded fairly well to the target chemical composition. This fact confirms the need to use physical synthesis methods for the production of Cu–Au nanoparticles.
